# Pregnancy-related acute myocardial infarction: a review of the recent literature

**DOI:** 10.1007/s00392-021-01937-5

**Published:** 2021-09-12

**Authors:** Andrea Carlo Merlo, Gian Marco Rosa, Italo Porto

**Affiliations:** 1 Department of Internal Medicine and Medical Specialties (DIMI), Chair of Cardiovascular Diseases, University of Genoa, Genoa, Italy; 2 Cardiology Unit, DICATOV—Cardiothoracic and Vascular Department, IRCCS Ospedale Policlinico San Martino, Genoa, Italy

**Keywords:** Pregnancy, Myocardial infarction, Coronary artery dissection, Foetus, Ionising radiation

## Abstract

Pregnancy-related acute myocardial infarction is a rare and potentially life-threatening cardiovascular event, the incidence of which is growing due to the heightened prevalence of several risk factors, including increased maternal age. Its main aetiology is spontaneous coronary artery dissection, which particularly occurs in pregnancy and may engender severe clinical scenarios. Therefore, despite frequently atypical and deceptive presentations, early recognition of such a dangerous complication of gestation is paramount. Notwithstanding diagnostic and therapeutic improvements, pregnancy-related acute myocardial infarction often carries unfavourable outcomes, as emergent management is difficult owing to significant limitations in the use of ionising radiation—e.g. during coronary angiography, potentially harmful to the foetus even at low doses. Notably, however, maternal mortality has steadily decreased in recent decades, indicating enhanced awareness and major medical advances in this field. In our paper, we review the recent literature on pregnancy-related acute myocardial infarction and highlight the key points in its management.

## Introduction

Cardiovascular diseases rank as the leading cause of pregnancy-related deaths in the United States, accounting for more than 15% of such deaths in the recent years, as reported by the Centers for Disease Control and Prevention. Notoriously, gestation heightens the risk of acute myocardial infarction (AMI) about threefold, owing to its associated hypercoagulability and hypervolaemia, which in turn increase heart rate, cardiac output, blood pressure, and myocardial oxygen consumption [1–3]. Moreover, according to recent evidence, pregnancy-related AMI and cardiovascular mortality are growing worldwide [4].

Over the last few decades, gestation above 35 years of age has undoubtedly become more frequent, as a result not only of social and economic changes (prolonged education, difficulty in finding employment, etc.), but also of the advent of assisted reproductive technology, which allows couples who were previously considered infertile to have children, and which is increasingly utilised [5]. Although the recent literature has excluded a causal link between successful fertility therapy and cardiovascular diseases, the extended childbearing potential of women may primarily contribute to the rising incidence of pregnancy-related AMI [5, 6]. Unfortunately, despite significant breakthroughs in cardiovascular medicine, this condition still results in poor maternal and foetal outcomes.

## Epidemiology

**Fig. 1:**
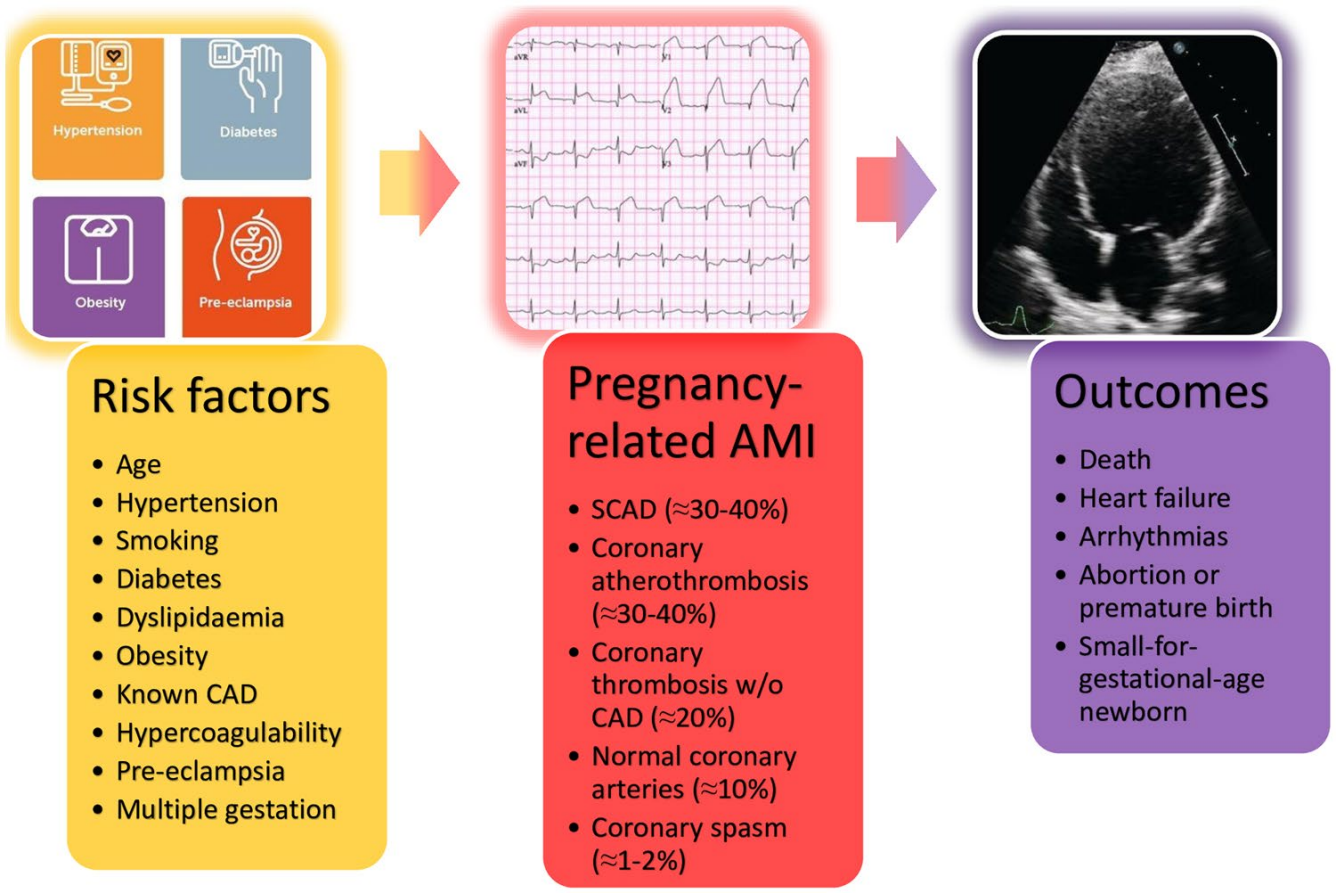
Progression from high cardiovascular risk to pregnancy-related AMI (with percent frequency of each aetiology) and its potentially severe outcomes for both mother and child. *AMI* acute myocardial infarction, *CAD* coronary artery disease, *SCAD* spontaneous coronary artery dissection

Despite rising maternal age, pregnancy-related AMI remains uncommon, its incidence varying between 0.06 and 10/100,000 worldwide [7, 8]. Indeed, although the risk of acute coronary events increases considerably with age, pregnancy-related AMI is a multifactorial disease that results from several predisposing factors and is only partly attributable to older age (Fig. 1) [4].

First, modifiable and non-modifiable—e.g. ethnicity and thrombophilia—cardiovascular risk profile plays a pivotal role in the pathogenesis of pregnancy-related AMI, just as it does in AMI in the general population. According to numerous studies, an increasing number of expectant mothers in the United States have chronic cardiovascular conditions, such as diabetes mellitus and ischaemic heart disease [9–15]. In this regard, the most prevalent risk factor is smoking (25% of cases), followed by hyperlipidaemia (20%) and hypertension (15%) [16]. In an observational retrospective study that analysed 55,402,290 hospitalisations during pregnancy or puerperium recorded in a United States national database, including 4471 cases of pregnancy-related AMI, women with AMI were older and more likely to have cardiovascular risk factors than those without [17]. In addition, known coronary artery disease (CAD) emerged as the strongest predictor of pregnancy-related AMI in a recent large observational study of an American population [4]. Nevertheless, according to the MBRRACE-UK report, 90% of pregnant women who died between 2014 and 2016 did not have a pre-existing cardiac condition, implying that most of the observed mortality might depend on acquired cardiovascular diseases [18].

Concerning obstetric conditions, multiple gestation, gestational diabetes, and hypertensive disorders of pregnancy (such as pre-eclampsia) appear to be the most relevant, whereas the contribution of haemorrhage and anaesthesia complications to deaths has declined [13]. Specifically, 18.3% of gestating women with AMI experienced some type of eclampsia/pre-eclampsia in one study [4]. Moreover, hypertensive disorders of pregnancy show an increasing trend, potentially leading to a higher incidence of CAD in the near future [19, 20].

In addition, modifications of hormonal levels, together with dysregulation of coagulation and fibrinolysis, may exacerbate the risk of thromboembolism [21].

Albeit traditionally reported to have a higher incidence in the peripartum period, pregnancy-related AMI may present at any stage of gestation, according to one meta-analysis [8]. In any case, multigravidas have a considerably higher risk, and prognosis is worse for events occurring peripartum [22].

## Pathogenesis

Pregnancy-related AMI may have several aetiologies. Spontaneous coronary artery dissection (SCAD) plays a major role, accounting for up to 43% of cases [16]. CAD seems to be the second leading cause, according to the recent literature [23, 24]. Coronary thrombosis and spasm in the absence of CAD are fairly rare aetiologies [16].

In general, SCAD predominantly affects young white females (83%), and one study showed no significant difference in the baseline characteristics between postpartum and non-postpartum patients with SCAD [25]. Like coronary spasm, SCAD mostly occurs postpartum in multiparous women, the average gravidity being 2.7. When antepartum, it mainly presents in the third trimester [26]. The timing of SCAD might be related to cardiac stress secondary to rapid post-delivery uterine contraction and the return of a copious volume of blood to the systemic circulation [27]. Physiological changes associated with pregnancy and the haemodynamic effects of labour can also precipitate SCAD, especially when predisposing conditions such as fibromuscular dysplasia coexist [28]. Mechanisms such as twin gestation and pre-existing or gestational hypertension may increase endothelial dysfunction and shear stress, thus promoting the onset of SCAD and acute aortic dissection [20, 29]. Conversely, the prevalence of conventional risk factors such as diabetes mellitus and chronic kidney disease is significantly lower in pregnant women with SCAD, with up to 61% of such patients not presenting any [16, 26]. It appears that an excess of progesterone can determine degeneration of elastic fibres and a decrease in acid mucopolysaccharide ground substance, whereas oestrogen may increase the release of matrix metalloproteinase [30, 31]. In addition, a large percentage of non-pregnant women affected by SCAD take oral contraceptives, which supports this observation [32]. The left anterior descending artery is more often involved, probably owing to haemodynamic differences among the coronary arteries [33]. Interestingly, despite limitations related to the small number of cases and possible selection bias, a cohort analysis of the Mayo Clinic SCAD Registry found no statistically significant association of SCAD recurrence with pregnancy among women with prior SCAD, suggesting that such an event is multifactorial with contributory causes beyond pregnancy alone [34].

Pregnancy-related AMI due to CAD shows a fairly uniform incidence during gestation [16]. In a European registry analysis of pregnant women and cardiovascular diseases, CAD was reported to occur even more commonly than SCAD [35].

Coronary thrombosis with no evidence of CAD causes a relatively high proportion of pregnancy-related AMIs, though it is very rare in the general population. This probably results from the above-mentioned hypercoagulability due to alterations in the coagulation and fibrinolytic systems, even if it may also follow direct or paradoxical embolism [36, 37].

Regarding coronary spasm, pre-eclampsia is a strong risk factor, as it causes systemic endothelial dysfunction owing to imbalance in the secretion of endothelin and thromboxane [38]. Other possible mechanisms in pregnancy include enhanced vascular reactivity to angiotensin II and noradrenaline, and renin and angiotensin release because of decreased uterine perfusion in the supine position [2].

However, it is generally difficult to identify the AMI phenotype in young women. Indeed, in one study, the mechanism of AMI, based on the current Universal Definition of myocardial infarction, was undetermined in 10% of patients [39].

## Diagnosis

The diagnostic criteria of pregnancy-related AMI are the same as in the general population, but non-invasive methods should generally be preferred in order to preserve foetal health [40].

The patient’s epidemiological profile is paramount, as the risk of AMI is particularly associated with older age, multiple gestation, and African ethnicity [7]. As often happens in young women, the clinical presentation may be atypical, including angina equivalents instead of chest pain, so that the arrival at the emergency department is frequently delayed [41]. Therefore, a low threshold should be applied to thoroughly investigate women with suspected acute myocardial ischaemia [23, 42].

The electrocardiogram (ECG) shows ST-segment elevation in about half of cases [16, 17]. Nonetheless, the relatively horizontal orientation of the heart during gestation limits the specificity of some ECG findings, which may also be present in healthy pregnant women. For example, moderate left axis deviation, T-wave inversion in leads V1–V2, and small Q waves together with inverted T waves in lead III are rather common. Changes mimicking left ventricular hypertrophy can occur. Furthermore, ST-segment depression simulating myocardial ischaemia has been observed after the induction of anaesthesia for caesarean section (CS) [43, 44].

Transthoracic echocardiography (TTE) may help detect acute myocardial ischaemia when the ECG is non-diagnostic, though its accuracy in pregnant women is usually lower.

Cardiac troponin (cTn) is the only specific biomarker, since creatine kinase MB and myoglobin may be heightened by uterine contraction during labour and delivery [45]. However, other conditions, such as pre-eclampsia and gestational hypertension, sometimes increase cTn plasma levels, thus compelling physicians to rule out pregnancy-related AMI [44].

When the diagnosis remains unclear, especially if cTn levels are normal or borderline, a submaximal ECG stress test may be carefully performed, however, this should be carried out under continuous foetal monitoring, owing to the risk of foetal bradycardia [40]. Alternatively, physical or pharmacological stress TTE is an option, as no adverse effects have been reported [46].

The use of computed tomography angiography (CTA) during pregnancy is controversial. In experienced centres, the radiation dose administered during CTA may be slightly lower than that delivered during coronary angiography (CA) [47]. However, when invasive management has been planned, CTA may determine a delay in treatment, thereby increasing maternal and foetal exposure [48].

Although imaging body regions outside the abdomen or pelvis usually exposes the uterus to a negligible radiation dose, CA should be avoided in pregnant patients unless high-risk features are present and an interventional strategy appears plausible. Foetal exposure during CA can be reduced by adopting a trans-radial approach, shortening fluoroscopy time, and ensuring appropriate abdominal protection [40]. When CA is deemed mandatory, the radiation dose must be kept “as low as reasonably achievable”, and preferably  < 50 mGy [41, 44]. Diagnostic CA exposes the foetus to less than 1.5 mGy, while foetal exposure during percutaneous coronary intervention (PCI) is slightly greater [44]. Doses between 50 and 100 mGy are regarded as inconclusive in terms of impact on the foetus [49]. Observed radiation-induced abnormalities (typically at doses of 100–200 mGy) include growth restriction, intellectual disability, malignancies, and neurological effects [50]. In general, termination of pregnancy is recommended if the estimated foetal radiation dose is  > 150 mGy, viewed as the minimum amount of dosage at which negative foetal consequences will occur [51]. The harmful effects of radiation on the foetus are also a function of gestational age, as the most at-risk period is between 2 and 7 weeks, when organogenesis takes place. In general, the foetus is more resistant to radiation during the second and third trimester than during the first one, even though radiologic exams ought to be avoided during the whole pregnancy unless strictly recommended [49]. Obviously, these considerations do not apply in the postpartum period, when no additional risk results from ionising radiation compared to non-pregnant women.

If CA is non-diagnostic, especially in suspected SCAD, intracoronary imaging—particularly optical coherence tomography—may provide significant incremental value [52]. However, the risk–benefit ratio of any medical intervention beyond standard CA should carefully be considered in this population, given the danger of iatrogenic dissection [43].

Tools for cardiac risk stratification in pregnancy include Cardiac Disease in Pregnancy (CARPREG) I, CARPREG II, ZAHARA, and the modified World Health Organisation classification. Nevertheless, all these have been developed and utilised to assess outcomes in pregnant women with congenital heart disease, valve disease, and cardiomyopathy [53]. In contrast, instruments for evaluating ischaemic heart disease during gestation are still lacking [54].

## Differential diagnosis

In pregnant patients with chest pain, differential diagnosis mainly includes pulmonary embolism (PE), aortic dissection, and pre-eclampsia [44]. All of these clinical scenarios show the highest incidence between the last trimester and the postpartum, where a significant proportion of coronary events occur as well. Moreover, they may determine an increase in cTn levels depending on several factors, which makes the diagnosis even more challenging [44]. Particularly, pregnancy increases the risk of PE about fourfold, compelling physicians to always rule it out [55]. Due to its epidemiological importance, an assessment of risk factors for venous thromboembolism is recommended to all women before pregnancy or in the first weeks of gestation [44].

Common clinical features of PE are pleuritic chest pain, dyspnoea, haemoptysis, palpitation, possibly associated with limb pain and/or swelling. However, the majority of such symptoms and signs may be simply related to physiological gestation, making the diagnosis challenging [50, 56]. The ECG may reveal sinus tachycardia or tachyarrhythmias, the S1Q3T3 sign, and right ventricular strain, whilst TTE may detect right ventricular dilatation and increased arterial pulmonary pressures, but the accuracy of these tests is lower in pregnant patients. The use of Wells score and D-dimer plasma levels, generally pivotal to estimate the probability of PE, has not been validated in pregnancy. Therefore, the optimal diagnostic approach for these patients remains currently uncertain [57]. Venous ultrasound sometimes demonstrates deep vein thrombosis, whereas magnetic resonance imaging may sufficiently explore the pulmonary circulation. Nonetheless, low-dose CTA should be performed if the clinical suspicion remains high once the above-mentioned investigations have not corroborated the diagnosis [44].

## Therapy

In general, close monitoring of the mother and the foetus is required, with a delivery strategy in place in case of maternal or foetal deterioration. In the event of maternal cardiac arrest, resuscitation and delivery should be performed according to existing guidelines [41, 58].

Available data on foetal tolerance of AMI pharmacotherapy are still scarce [59]. Low-dose aspirin seems to be safe, but we have almost no information about the use of P2Y_12_ inhibitors, bivalirudin, and glycoprotein IIb/IIIa inhibitors. Therefore, clopidogrel is recommended solely when strictly necessary and for the shortest duration, whilst other antithrombotic drugs are generally contraindicated [16, 41]. The use of unfractionated heparin (UFH), enoxaparin, and fondaparinux is reasonable after careful evaluation of the bleeding risk. Specifically, predisposition to bleeding is highest in puerperium patients, and postpartum haemorrhage may arise up to 12 weeks after birth by definition, thus requiring a cautious management of antithrombotic therapy both in the acute and chronic setting. Particularly, compared with other drugs, a careful peripartum management of anticoagulants is compelling for physicians, in order to lower the risk of postpartum haemorrhage. UFH usually constitutes the first choice in pregnancy, given its early-onset effect, short half-life, and easy dose adjustment through close monitoring of the activated clotting time [60, 61]. Moreover, UFH does not cross the placenta and does not cause foetal bleeding or malformations. Importantly, UFH can be effectively antagonised by protamine sulphate if prompt reversal of anticoagulation is necessary—e.g. around the time of delivery. In this regard, it is usually deemed safe to switch low-molecular-weight heparin to UFH and stop UFH administration 4–6 h before delivery, resuming it 6 and 12 h after vaginal birth and CS, respectively, if no bleeding complications occur [44]. However, despite the development of heparin-induced thrombocytopenia in 3% of patients, benefits of UFH significantly outweigh risks in most clinical scenarios, including CA for the diagnosis of acute coronary syndromes [60, 61]. Contrariwise, low-dose aspirin is never a concern for bleeding during pregnancy.

Beta-blockers can help reduce both myocardial oxygen consumption and shear stress in SCAD. They are well-tolerated and widely utilised in thyrotoxicosis, hypertension, hypertrophic cardiomyopathy, and arrhythmias during pregnancy, particularly the cardio-selective ones [40].

Calcium-channel blockers seem to be safe in pregnancy, though they are never first-line drugs.

Angiotensin-converting enzyme inhibitors, angiotensin receptor blockers, and statins are contraindicated, owing to their potentially harmful effects on the foetus [41].

For what concerns revascularisation, the current European Society of Cardiology (ESC) guidelines endorse a conservative management in stable, low-risk cases of pregnancy-related AMI. Conversely, in cases presenting ST-segment elevation and/or other high-risk criteria, prompt invasive management, possibly followed by primary PCI, should be undertaken [41]. Owing to the pregnancy-related bleeding risk—e.g. during delivery—the duration of post-PCI dual antiplatelet therapy with second/third-generation drug-eluting stents (DES) can be shortened, especially in the absence of a high thrombotic burden [41]. Despite general preference for the use of DES rather than bare-metal stents (BMS) in patients with acute coronary syndromes supported by the ESC guidelines, most reports on AMI in young and pregnant women regard BMS, whereas evidence concerning the safety and benefits of DES in such patients is anecdotal [44, 62, 63]. Therefore, according to some authors, BMS in place of DES could be considered in selected cases [43]. Nevertheless, in our opinion, given the very low incidence of stent thrombosis as well as the safety of shortening the duration of dual antiplatelet therapy following implantation of last-generation DES, even in this subpopulation the choice of BMS rather than DES appears no more reasonable.

Coronary artery bypass grafting ought to be reserved for cases complicated by failure of PCI, as it usually has unfavourable outcomes [28]. Specifically, foetal mortality is reported to be as high as 14–33% [64].

Thrombolysis should never be performed during pregnancy and peripartum except for high-risk PE—e.g. complicated by severe haemodynamic instability—as the bleeding risk related to it is deemed too high [65]. Nonetheless, thrombolytics do not significantly cross the placenta and they do not have teratogenic effects. When thrombolysis is necessary, the loading dose of UFH ought not to be given [44].

**Fig. 2:**
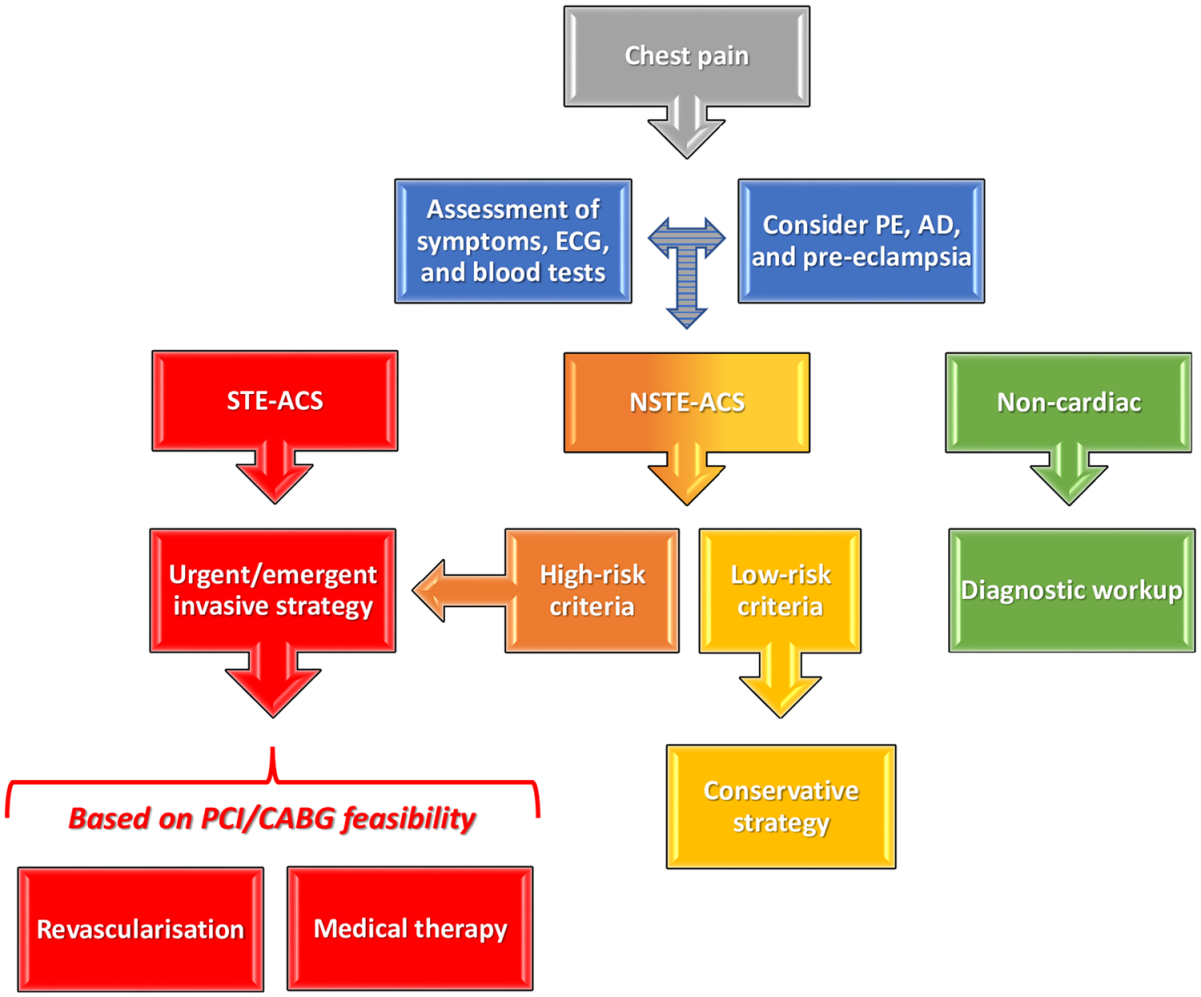
Practical management of chest pain in a pregnant woman according to current guidelines. *ACS* acute coronary syndrome, *AD* aortic dissection, *CABG* coronary artery bypass graft, *ECG* electrocardiogram, *NSTE* non-ST elevation, *PCI* percutaneous coronary intervention, *PE* pulmonary embolism, *STE* ST elevation

A practical management of suspected pregnancy-related AMI as per current guidelines is shown in Fig. 2.

## Outcomes

Unfortunately, more than half of pregnancy-related AMIs are followed by a permanent reduction in left ventricular ejection fraction; this reflects serious myocardial damage to the anterior left ventricular wall, which is associated with a high incidence of life-threatening complications and death [16, 66]. Some studies have reported that women with SCAD during pregnancy have a poorer prognosis than those with SCAD unrelated to gestation [27, 67].

Nevertheless, global mortality has shown a decreasing trend since the 1980s, improving from 37 and 21% in 1985 and 1996, respectively, to 7.3% in the 1990s and 5.1% in 2000–2002 [66, 68–70]. This probably follows significant improvements in diagnosis and treatment over time and bias in collecting data mainly from severe or fatal cases in older reports. Accordingly, in the largest prospective study to date on pregnant women affected by acute coronary syndromes, no maternal deaths were recorded [35]. In line with this result, another recent study observed comparable rates of in-hospital mortality after AMI between pregnant women and the general population. Consistently with other previous reports, most major adverse cardiovascular and cerebrovascular events occurred in the postpartum period [4].

Despite the relatively low foetal mortality in pregnancy-related AMI, the incidence of preterm birth and neonatal death remains high. Nonetheless, in contrast with reports regarding women with non-ischaemic heart disease, the frequency of small for gestational age newborns was not elevated in one study [71].

Moreover, the recent literature suggests that rates of CS are notably higher in women with pregnancy-related AMI, more than twice those in healthy women in Europe [35, 72]. Indeed, CS appears to be the safest approach to delivery after an acute cardiac event. However, women with preserved cardiac function can face vaginal delivery, which should usually be preferred for both mother and child, particularly since the haemodynamic stress secondary to labour and birth can be reduced by epidural anaesthesia and instrumental assistance. Indeed, CS is recommended mainly for high-risk cases, e.g. after a recent AMI or if a reduced left ventricular ejection fraction persists. In general, however, delivery should be delayed for a few weeks after AMI, to reduce haemodynamic stress immediately after the event [44].

## Conclusions

Pregnancy heightens the risk of AMI, and its incidence is poised to grow as maternal age increases. However, an interplay of modifiable and non-modifiable cardiovascular risk factors, obstetric conditions, and pre-existing cardiac disease has a pivotal role and can lead to adverse outcomes. Compared with healthy pregnant women, those with AMI are more often smokers, hypertensive, overweight, and diabetic. Therefore, cardiovascular prevention through lifestyle modification and risk factor control should be strongly recommended for both pregnant women and those planning to become pregnant. As for SCAD recurrence during gestation, recent data seem to be reassuring but require prudent interpretation.

Despite frequently atypical presentations, physicians should seriously consider the diagnosis of pregnancy-related AMI when an expectant mother presents with chest pain or an angina equivalent. Although most cases should be managed conservatively, high-risk features must prompt an urgent invasive approach.

Pregnancy-related AMI traditionally carries a poor prognosis for both mother and child. Particularly, premature birth and CS rates are increased in affected women. However, the recent literature shows that maternal mortality and the incidence of complications have significantly decreased over the last few decades, probably owing to improved diagnosis and treatment.

Numerous gaps in our knowledge of pregnancy-related AMI remain, largely due to the rarity of the condition and the paucity of data. Consequently, big studies are required to better understand its causes and triggers and inform therapy.
